# Dispersal of male and female *Culex quinquefasciatus* and *Aedes albopictus* mosquitoes using stable isotope enrichment

**DOI:** 10.1371/journal.pntd.0005347

**Published:** 2017-01-30

**Authors:** Matthew C. I. Medeiros, Emily C. Boothe, E. Brendan Roark, Gabriel L. Hamer

**Affiliations:** 1 Department of Entomology, Texas A&M University, College Station, Texas, United States of America; 2 Pacific Biosciences Research Center, University of Hawai‘i at Mānoa, Honolulu, HI, United States of America; 3 Department of Geography, Texas A&M University, College Station, Texas, United States of America; University of Florida, UNITED STATES

## Abstract

The dispersal patterns of mosquito vectors are important drivers of vector-borne infectious disease dynamics and understanding movement patterns is pivotal to devise successful intervention strategies. Here, we investigate the dispersal patterns of two globally important mosquito vectors, *Aedes albopictus* and *Culex quinquefasciatus*, by marking naturally-occurring larvae with stable isotopes (^13^C or ^15^N). Marked individuals were captured with 32 CDC light trap, 32 gravid trap, and 16 BG Sentinel at different locations within two-kilometer radii of six larval habitats enriched with either ^13^C or ^15^N. In total, 720 trap nights from July to August 2013 yielded a total of 32,140 *Cx*. *quinquefasciatus* and 7,722 *Ae*. *albopictus*. Overall, 69 marked female mosquitoes and 24 marked male mosquitoes were captured throughout the study period. The distance that *Cx*. *quinquefasciatus* females traveled differed for host-seeking and oviposition-seeking traps, with females seeking oviposition sites traveling further than those seeking hosts. Our analysis suggests that 41% of *Cx*. *quinquefasciatus* females that were host-seeking occurred 1–2 kilometer from their respective natal site, while 59% remained within a kilometer of their natal site. In contrast, 59% of *Cx*. *quinquefasciatus* females that were seeking oviposition sites occurred between 1–2 kilometer away from their larval habitat, while 15% occurred > 2 kilometer away from their natal site. Our analysis estimated that approximately 100% of *Ae*. *albopictus* females remained within 1 km of their respective natal site, with 79% occurring within 250m. In addition, we found that male *Ae*. *albopictus* dispersed farther than females, suggesting male-biased dispersal in this *Ae*. *albopictus* population. This study provides important insights on the dispersal patterns of two globally relevant vector species, and will be important in planning next generation vector control strategies that mitigate mosquito-borne disease through sterile insect techniques, novel *Wolbachia* infection, and gene drive strategies.

## Author summary

Resolving patterns of mosquito dispersal across landscapes is a critical step toward the development of effective control strategies that mitigate vector-borne disease transmission and its public health burden. Here, we used a recently developed technique involving the enrichment of aquatic larval habitat with stable isotopes to mark male and female mosquitoes of two important vector species, *Aedes albopictus* and *Culex quinquefasciatus*. We show that the patterns of dispersal were fundamentally different between these urban vectors. *Culex quinquefasciatus* dispersed much further than *Aedes albopictus*. In addition, male *Aedes albopictus* dispersed further than female mosquitoes. Our study suggests that infectious disease agents transmitted by *Culex quinquefasciatus* will be more difficult to control locally than those transmitted by *Aedes albopictus*. Our results on sex-biased dispersal in *Aedes albopictus* have implications for next-generation vector control strategies that rely on the release of sterile or sterilizing males to control mosquito populations. Finally, our study continues to show the utility of the stable-isotope marking technique to study mosquito movement.

## Introduction

Understanding patterns of mosquito dispersal is paramount to the development of effective vector control strategies that mitigate vector-borne disease transmission and its public health burden [1]. The spatial scales of vector control techniques are optimized with information on adult mosquito dispersal. For instance, programs that utilize insecticides must calibrate the spatial scale of insecticide treatment to adult dispersal distances for maximal success [2, 3]. The field implementation of next generation vector control technologies that involve the release of autocidal, sterile, or *Wolbachia*-infected individuals must account for the likely dispersal distance of released individuals [4, 5], and adjust the number and geographic spread of release sites to cover target areas. At larger scales, mosquito dispersal may facilitate the spread of infectious diseases into new regions, shaping geographical patterns of disease emergence and our ability to predict and contain the spread of infectious agents [6].

Mosquitoes disperse to find resting sites, mates, nectar sources, blood sources, and oviposition sites [7, 8]. The distribution of these resources in the environment is an important modulator of mosquito movement. For instance, Edman et al. [9] demonstrated that the probability of dispersal of female *Aedes aegypti* (Linnaeus) is greater from homes that have fewer potential oviposition sites. Maciel-De-Freitas et al. [10] demonstrated that *Aedes albopictus* (Skuse) released in forests preferentially dispersed over a kilometer to urban areas to feed on humans, while those released within urban environments were generally sedentary. Additionally, patterns of dispersal may vary between species in the same environment. Many studies suggest that *Ae*. *aegypti* [11–14] and *Ae*. *albopictus* [15–18] are short dispersing mosquitoes, generally moving less than a few hundred meters. In contrast, other vectors like *Culex pipiens* (Linnaeus) [19] and *Cx*. *quinquefasciatus* (Say) [20–23] mosquitoes may disperse several kilometers. Landscape features can impact mosquito dispersal patterns by serving as corridors that promote mosquito movement. In addition, weather conditions also impact mosquito dispersal distance and direction. For instance, Lapointe [21] demonstrated that *Cx*. *quinquefasciatus* in Hawaii moved along roads in the landscape and dispersed predominately in the direction of prevailing winds. Finally, some studies have suggested that mosquito dispersal patterns vary between sexes [24], while others suggest that they are generally similar [25, 26].

Dispersal in mosquitoes has been investigated using mark-release-recapture designs in which adult mosquitoes are marked with dusts, dyes, paints, trace elements, and radioactive isotopes [27]. Typically, these marked adult individuals are released at a specific point and then subsequently recaptured at other sites. These techniques can be highly invasive, tedious, and require the rearing and marking of large quantities of adults. While utilized widely, these methods may alter the behavior of the mosquito, introducing artifacts in data that skew dispersal patterns. In addition, the artificial release of these insects augments local populations, potentially increasing the capacity for those populations to transmit infectious disease. Ideal protocols for adult mosquito mark-recapture studies should involve a marker that is environmentally safe, cost-effective, easy to use, and does not inhibit normal vector biology [27].

Stable isotopes offer safe and useful biological tracers as they occur naturally in the environment, do not decay, and are non-toxic. In a recent study, Hamer et al. [28] developed a stable isotope method to mark naturally-occurring mosquitoes during their larval stage. The laboratory experiments from this study suggested life-long retention of the marker with no apparent impact on morphology or survival. There are several advantages to using stable isotopes to mark mosquitoes during dispersal studies. One principal advantage is the ability to mark naturally occurring immature mosquitoes in aquatic habitat by treatment with isotopically-enriched material. The larval habitat of mosquitoes, such as *Culex* sp. and *Aedes* sp., is typically confined (often in artificial containers in urban settings) and thus easily enriched with stable isotopes during larval development. Hamer et al. [28] also showed that there is no evidence of transgenerational marking and that the isotopic retention was higher in ^15^N-enriched adults (δ^15^N = +500 ‰ at 55 days post emergence) than ^13^C-enriched mosquitoes (δ^13^C = +100 ‰ at 55 days post emergence). Hamer et al. [19] implemented this method to study the dispersal of *Cx*. *pipiens*, the primary vector for West Nile virus (WNV), in suburban Chicago, Illinois. That study enriched *Culex* larvae in productive catch basins with ^15^N. A survey of over 30,000 mosquitoes revealed 12 ^15^N-marked individuals, yielding a mean distance traveled of 1.15km.

Here, we use a similar stable isotope marking protocol to investigate the dispersal of two important mosquito vectors, *Cx*. *quinquefasciatus* and *Ae*. *albopictus*, in central Texas, a severely neglected arbovirus hotspot [29]. *Cx*. *quinquefasciatus* is accepted as the principal vector of WNV in much of the southern United States [30–32], and is a globally important vector of human filariasis [33], St. Louis encephalitis virus [34], and avian malaria [35]. *Ae*. *albopictus* is now a globally invasive mosquito that originated in southeast Asia. This species is a competent vector for numerous arboviruses, including dengue virus (DENV) [36], chikungunya virus (CHIKV) [37–39], yellow fever virus (YFV) [40], and potentially Zika virus (ZIKV) [41], which has recently emerged across the Americas. Given the cosmopolitan distributions of *Cx*. *quinquefasciatus* and *Ae*. *albopictus*, an improved understanding of dispersal behavior and distance will improve the management of these important vectors globally.

## Materials and methods

### Stable isotope enrichment

From July 1^st^ to August 31^st^ 2013, two sites were treated with stable isotopes, one site with ^15^N-potassium nitrate (30° 36' 16.83"N, 96° 19' 34.29"W) and the other site with ^13^C-glucose (30° 36' 11.196"N, 96° 19' 48.021"W). Enrichment sites were separated by approximately 0.5km and each site consisted of three black tubs (i.e. artificial containers), 30 (width) x 50 (length) by 20 cm (height), filled with approximately three liters of water, and allowed mosquitoes to naturally develop within the environment. The initial treatment concentration was 2.0 mg of isotope per liter of water. Every third week, one container from each enrichment site was disposed of; new water was added and again enriched with the initial treatment. *Culex* egg rafts from wild females were laid directly on the water and a Whatman filter paper was taped to the side of the tub to allow *Ae*. *albopictus* oviposition. The filter paper containing *Aedes* eggs were dried for 48 hours and then submerged in the tub water to allow hatching. Because the larval habitats were confined, there was no concern of downstream enrichment of the surrounding environment. The containers were consistently monitored for any evaporation, exploitation, and rainfall events causing overflow. Containers were inspected for egg rafts, larvae, and pupae every three days, under the assumption that there would be new pupae every 48-72h. We used the counts of pupae to estimate the number of mosquitoes of each species emerging from enriched containers over the study period. Subsamples of 10-4^th^ instars and pupae were collected and identified as *Cx*. *quinquefasciatus* or *Ae*. *albopictus*, and were submitted for stable isotope analysis to confirm enrichment.

### Larvicide

During the mark-capture study, we implemented a larvicide program by treating larval habitat of *Culex* and *Aedes* mosquitoes with methoprene products (Altosid). One justification for larvicide was to ensure that our project would not result in a net increase in mosquito productivity in a region with a potential for arbovirus transmission. Even though we were allowing a few containers to produce mosquitoes, we would remove many more mosquitoes from the landscape by implementing a larvicide program in addition to our intensive adult mosquito trapping. The second justification for conducting a larvicide program was to reduce the total un-marked mosquito population in the study region. With this larvicide program we increased the probability of detecting an isotopically-enriched pool (i.e. marked pool) by reducing the population of un-marked mosquitoes in the study area.

Prior to the start of the stable isotope amendment, we surveyed containers in the mark-capture study region (213.5 hectares) for the presence of water and immature mosquitoes. On June 26th and July 12th 2013, habitat containers containing water were treated with either a Altosid 7-gram water-soluble packet (30-day submerged residual activity; 4.25% S-methoprene) or 3.5-grams of the granular formula (up to 21-day residual control; 4.25% S-methoprene). The stagnant margins of a 1.1 km creek running through the residential study area was also treated with Altosid extended release briquette (150-day residual control; 2.1% S-methoprene). A combination of 14 briquettes, 425 g of the granular formula and 53 water-soluble packets were distributed to various containers holding water and the creek on June 26, 2013. On July 12, 2013, these same habitat containers and the creek were treated again with 6 briquettes, 1,000 g of granular formula and 133 water-soluble packets. Water habitat was treated with these various products of different formulations according to the Altosid label. The active ingredient in Altosid, *S*-methoprene is an insect growth inhibitor that does not influence the oviposition behavior of *Culex* and *Aedes* mosquitoes [42, 43]. Therefore, it is unlikely that the Altosid treatment resulted in changes in female mosquito dispersal that is in part modulated by oviposition behavior.

### Adult mosquito trapping

**Fig 1:**
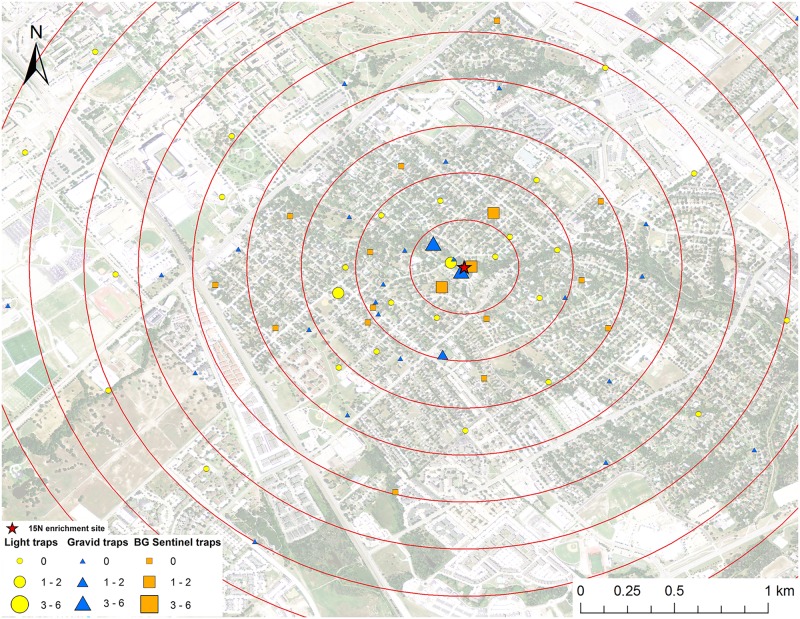
Map of mark-capture study region in College Station, Texas demonstrating the ^15^N-enrichment site and the spatial distribution of enriched female *Cx*. *quinquefasciatus* captures. Numbers next to symbols in legend represents the number of marked pools captured at each location. The satellite image was published by the USDA Aerial Photography Field Office as part of the National Agriculture Imagery Program (NAIP), and downloaded as a GIS file. The figure was produced using ArcGIS 10.2 (Esri, Redlands, CA).

**Fig 2:**
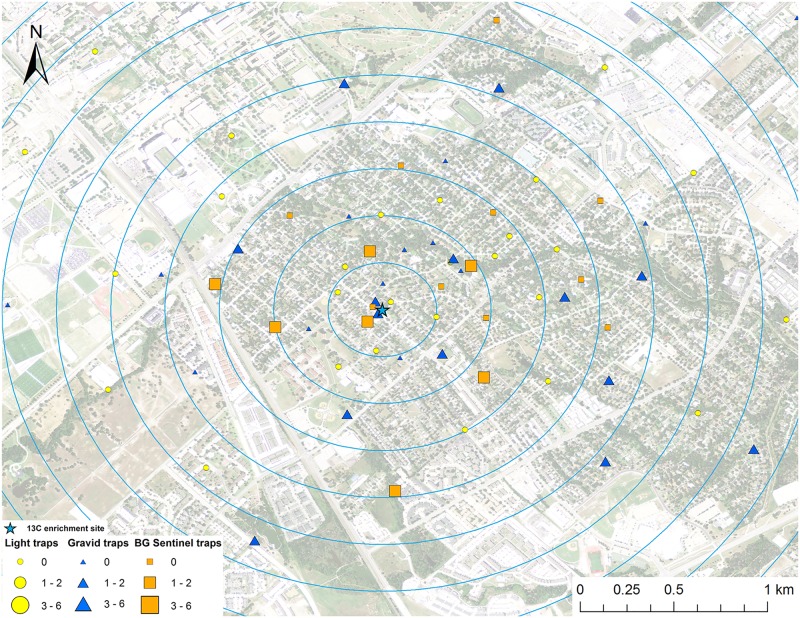
Map of mark-capture study region in College Station, Texas demonstrating the ^13^C-enrichment site and the spatial distribution of enriched female *Cx*. *quinquefasciatus* captures. Numbers next to symbols in legend represents the number of marked pools captured at each location. The map image was published by the USDA Aerial Photography Field Office as part of the National Agriculture Imagery Program (NAIP), and downloaded as a GIS file. Figure was produced using ArcGIS 10.2 (Esri, Redlands, CA).

**Fig 3:**
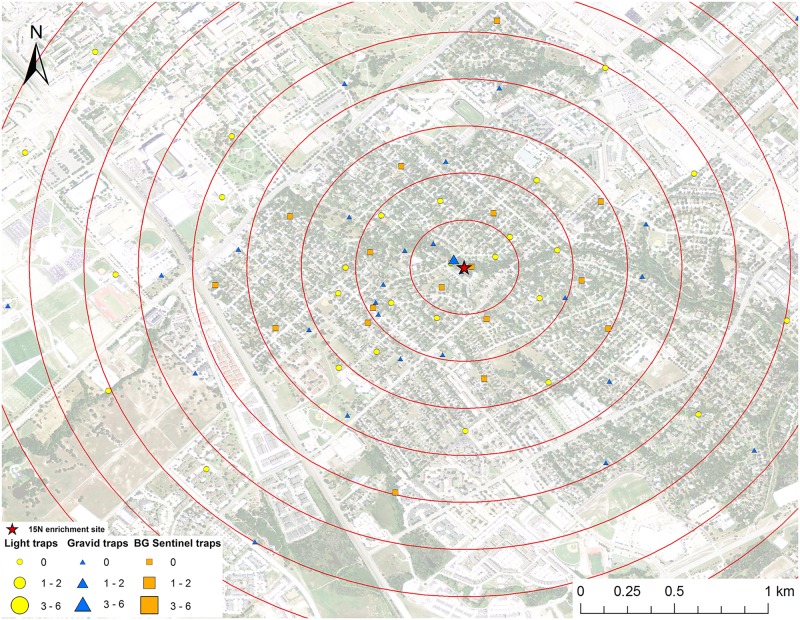
Map of mark-capture study region in College Station, Texas demonstrating the ^15^N-enrichment site and the spatial distribution of enriched male *Cx*. *quinquefasciatus* captures. Numbers next to symbols in legend represents the number of marked pools captured at each location. The map image was published by the USDA Aerial Photography Field Office as part of the National Agriculture Imagery Program (NAIP), and downloaded as a GIS file. Figure was produced using ArcGIS 10.2 (Esri, Redlands, CA).

**Fig 4:**
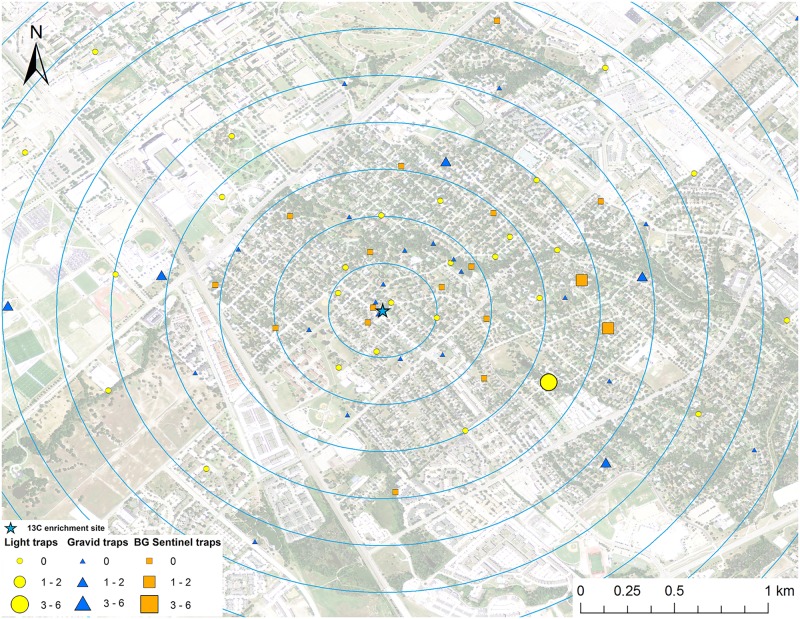
Map of mark-capture study region in College Station, Texas demonstrating the ^13^C-enrichment site and the spatial distribution of enriched male *Cx*. *quinquefasciatus* captures. Numbers next to symbols in legend represents the number of marked pools captured at each location. The map image was published by the USDA Aerial Photography Field Office as part of the National Agriculture Imagery Program (NAIP), and downloaded as a GIS file. Figure was produced using ArcGIS 10.2 (Esri, Redlands, CA).

**Fig 5:**
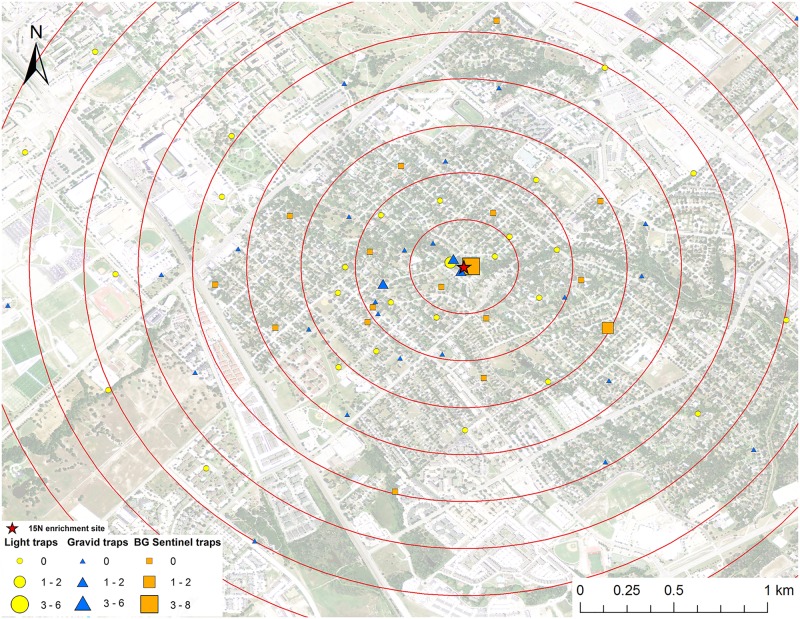
Map of mark-capture study region in College Station, Texas demonstrating the ^15^N-enrichment site and the spatial distribution of enriched female *Ae*. *albopictus* captures. Numbers next to symbols in legend represents the number of marked pools captured at each location. The map image was published by the USDA Aerial Photography Field Office as part of the National Agriculture Imagery Program (NAIP), and downloaded as a GIS file. Figure was produced using ArcGIS 10.2 (Esri, Redlands, CA).

**Fig 6:**
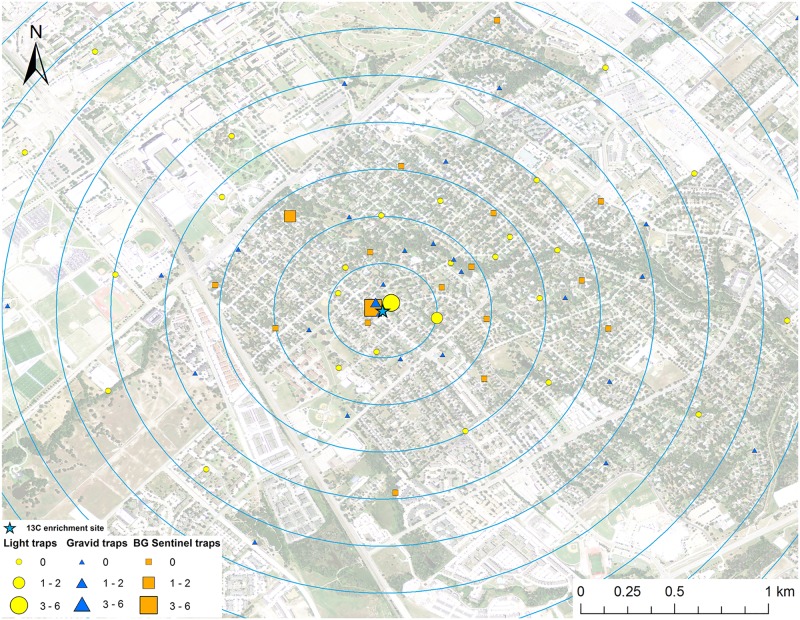
Map of mark-capture study region in College Station, Texas demonstrating the ^13^C-enrichment site and the spatial distribution of enriched female *Ae*. *albopictus* captures. Numbers next to symbols in legend represents the number of marked pools captured at each location. The map image was published by the USDA Aerial Photography Field Office as part of the National Agriculture Imagery Program (NAIP), and downloaded as a GIS file. Figure was produced using ArcGIS 10.2 (Esri, Redlands, CA).

**Fig 7:**
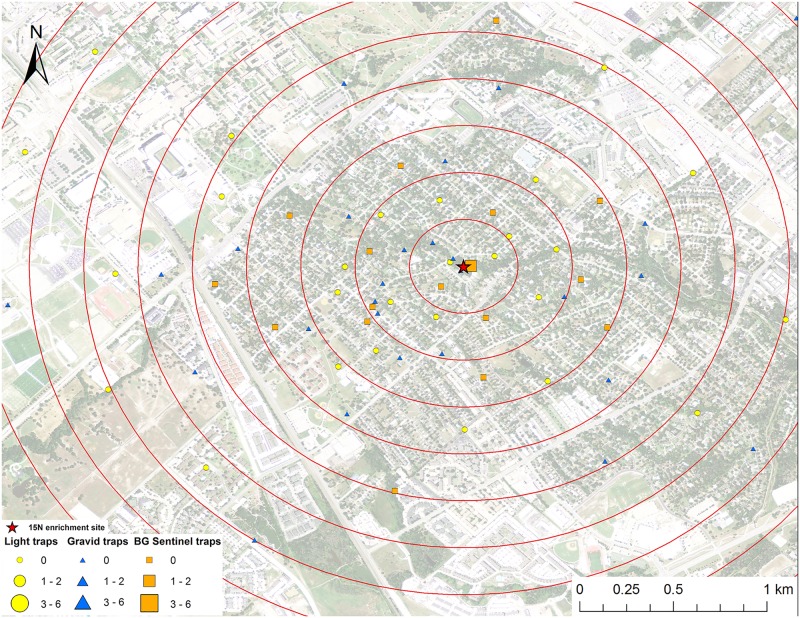
Map of mark-capture study region in College Station, Texas demonstrating the ^15^N-enrichment site and the spatial distribution of enriched male *Ae*. *albopictus* captures. Numbers next to symbols in legend represents the number of marked pools captured at each location. The map image was published by the USDA Aerial Photography Field Office as part of the National Agriculture Imagery Program (NAIP), and downloaded as a GIS file. Figure was produced using ArcGIS 10.2 (Esri, Redlands, CA).

**Fig 8:**
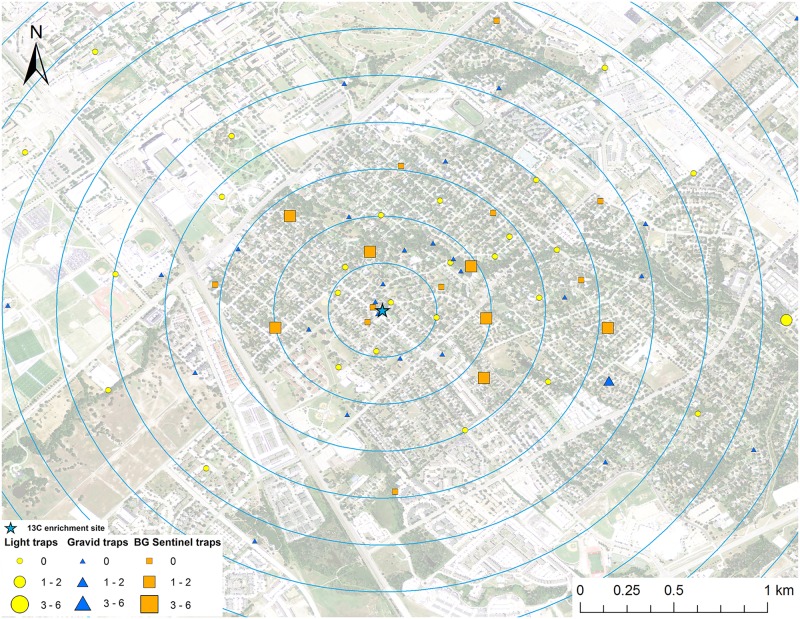
Map of mark-capture study region in College Station, Texas demonstrating the ^13^C-enrichment site and the spatial distribution of enriched male *Ae*. *albopictus* captures. Numbers next to symbols in legend represents the number of marked pools captured at each location. The map image was published by the USDA Aerial Photography Field Office as part of the National Agriculture Imagery Program (NAIP), and downloaded as a GIS file. Figure was produced using ArcGIS 10.2 (Esri, Redlands, CA).

Mosquitoes were trapped from May to September 2013 in College Station, Texas. Three types of mosquito traps were used for this experiment: 32 CDC gravid traps (The John Hock Company, Gainesville, Florida), 32 CDC miniature light traps (BioQuip Products, Inc., Rancho Dominguez, CA), and 16 BG-Sentinel traps (Biogents, Regensburg, Germany) were set weekly (Figs 1–8). Gravid traps were baited with organically enriched water made by infusing rabbit pellets with water, leaving in the sun for about six days, and then deploying in gravid trap tubs. Light and BG traps were baited with CO_2_ by placing a cooler with 2 kg of dry ice next to the traps. The BG traps were also baited with the BG-Lure. All traps were set in the evening and collected the next morning and each trap location was visited once per week during the study. Trap locations were placed in all directions from the enrichment sites (Figs 1–8), with a goal of an even distribution of the three trap types in the cardinal directions. The density of traps was highest closest to the release point and the exact location of traps was dictated by receiving permission from private homeowners. The closest mosquito trap was 26.6 m and the furthest was 2.16 km from the ^15^N enrichment site. The mosquito trap nearest to the ^13^C enrichment site was at 27.7 m and the furthest was 2.46 km away. The mean trap distance for ^13^C and ^15^N was 0.96 km and 0.95 km, respectively. Mosquitoes were identified to species and sex based on morphological keys [44, 45]. Approximately half of the individual female *Cx*. *quinquefasciatus* and *Ae*. *albopictus* mosquitoes were placed in pools of up to 4 individuals and prepared for stable isotope testing following the same general protocol of Hamer et al [28].

Weather data were collected using an existing weather station located at the Texas A&M University Turfgrass Research Laboratory approximately 3 km northwest from the ^13^C and ^15^N enrichment sites. The weather station recorded hourly data for temperature, wind speed, wind direction, and precipitation. From the weather observation data, East-West and North-South components of wind speed and direction vector were calculated for each month, and total season [19].

### Stable isotope analysis

Fourth instar larvae, pupae, and adult mosquitoes were stored at -40°C and processed for stable isotope analysis by drying and crimping each sample. Mass was estimated based on previously recorded data. Samples were dried at 50°C for 18–24 h, encapsulated into tin capsules that were crimped into a spherical shape, placed into a 96-well plate arranged to include standards, and submitted for stable isotope carbon (^13^C/^12^C) and nitrogen (^15^N/^14^N) analysis at the Stable Isotope Geosciences Facility, Texas A&M University, College Station, Texas. Initial samples, which required a shorter turn-around period in order to facilitate enrichment activities, were sent to Isotech Laboratories Inc., Champaign, Illinois. Texas A&M samples were analyzed using a Carlo Erba NA 1500 Series 2 Elemental Analyzer (EA) attached to a ThermoFinnigan Conflo III and a ThermoFinnigan Delta Plus XP isotope ratio mass spectrometer (IRMS). Briefly the EA combusts the samples at 1,200°C and the combusted samples passed through two reactors to convert the nitrogen oxides generated in the oxidation reactor to N_2_ gas. The resulting CO_2_ and N_2_ gases are chromatographically separated and then analyzed on the IRMS.

The peak areas of sample mass-to-charge ratios 28 (N_2_) and 44 (CO_2_) of a combusted sample are converted to total mass of nitrogen and carbon, respectively, using an intra-run calibration that allows for the calculation of the total nitrogen and carbon content. Raw sample δ^15^N and δ^13^C measurements are converted to the Air and VPDB isotopic scales, respectively, through an intra-run, two-point calibration of ~1 mg of L-glutamic acid standards with known isotopic values (USGS 40: δ^15^N = -4.52‰ Air, δ^13^C = -26.39‰ VPDB and USGS 4: δ^15^N = 47.57‰ Air, δ^13^C = 37.63‰ VPDB). Results are presented in standard delta (δ) notation: δX = [(Rsample/Rstandard)-1] x 1,000, where R is the ratio of the heavy to light stable isotope in the sample and standard. Internal laboratory standards (approximately every 12 unknowns) are used to measure analytical precision. For natural abundance samples, the analytical uncertainty is ± 0.20‰ for δ^13^C and δ^15^N. For labeled samples whose isotopic value extends beyond the range of the USGS 40 and 41 calibration standards the uncertainty will increase the more positive the value.

### Data analyses

To quantify Mean Distance Traveled (MDT) for each of our captured marked mosquito, we used a formula outlined by Silver [1] with a correction factor accounting for different trap densities in each annulus [19, 46]. We modeled the probability that species-specific pools composed of 2–4 female mosquitoes were marked by stable isotope enrichment with a general linear mixed model assuming a binomial error distribution. Models were implemented in the lme4 [47] package in program R. We added isotope (^15^N or ^13^C), week of capture (8 levels), and location of trap (78 groups) as random effects in the model. The number of individuals per pool was incorporated into the model by the offset function in program R. For *Cx*. *quinquefasciatus*, models included 4,098 observations. Fixed effects included distance from trap location, trap type (host seeking trap like a BG-Sentinel or CDC light trap, or an oviposition trap), direction of dispersal (four levels of 90° portions with due North set at 0° from source locations), and an interaction between distance and trap type. We discriminated on the basis of Akaike Information Criteria corrected for small sample size (AICc) among a set of candidate models including a full model with all fixed effects, 5 nested models of the full model, and an intercept only model. For *Ae*. *albopictus*, models included 1,372 observations. Given the small dispersal distances and lower recapture rate, we only included distance from larval habitat source as a fixed effect in a model and compared it to an intercept only model based on AICc.

We tested for differences between the sexes in the relationship between the probability of marked pools and distance from the larval source habitat with a sex*distance interaction. This modeling strategy also used a general linear mixed model assuming a binomial error distribution implemented in lme4 package, with the same random error structure described above. We tested the interaction effect with a parametric bootstrap of the log-likelihood ratio between a model that included the interaction in addition to the main effects and a model that included only the main effects. The bootstrap was implemented in the R package “pbkrtest” [48].

We estimated an index of relative density of marked mosquitoes within consecutive annuli centered on the enrichment site based on predictions from the best-fit models for each species, using modified equations from Morris et al [46]. We assume that model predictions of the probability of pool enrichment represent the probability of detecting a dispersing mosquito. The probability of detecting a dispersing individual is assumed to be directly proportional to the number of dispersing mosquitoes (i.e. marked mosquitoes) and the trap effort within an annulus (i.e. the number of traps operated across trap type), and inversely proportional to the area of the annulus (see Eq 1).

$$P_{Detection}\approx \frac{Number\;of\;dispersing\;mosquitoes\;\times \;Trap\;Effort}{Area}$$ (1)

We solve the equation for the estimated number of dispersing mosquitoes and interpret it as an index of the density of dispersing mosquitoes in our study. Relative density was estimated as the value for the number of dispersing mosquitoes of a given annulus divided by the sum of those values across all annuli.

## Results

### Stable isotope enrichment

Mosquito larvae were collected from treated containers and had a mean enrichment of 1,130.7±914.8 (n = 20) and 226.7±305.5 (n = 16) for δ^15^N and δ^13^C, respectively. We estimated that our larval habitats produced 1,240 *Cx*. *quinquefasciatus* and 1,003 *Ae*. *albopictus* from July 1^st^ to August 31^st^ 2013 by counting the number of pupae present in the enriched larval habitat every 48–72 hours. This number was used to estimate recapture rates (see below). Throughout the manuscript, we assume a 1:1 sex ratio in the adult population that emerged from the enriched larval habitat. A total of 298 and 482 larvae subsampled throughout the field season were collected and identified to be *Ae*. *albopictus* (^13^C n = 234, ^15^N n = 64) and *Cx*. *quinquefasciatus* (^13^C n = 157, ^15^N n = 325), respectively.

### Adult mosquito trapping

We collected a total of 71,962 female mosquitoes between May and September, of which 32,140 were *Cx*. *quinquefasciatus* (44.7%) and 7722 were *Ae*. *albopictus* (10.7%). A total of 2,758 female pools and 331 male samples were analyzed for the presence of enriched ^15^N and ^13^C. Of these, 69 (2.5%) and 24 (7.3%) female and male pools were enriched with a stable isotope, respectively. Eight enriched female and 2 male pools were captured in light traps, 29 female and 7 male pools were captured in gravid traps and 32 female and 15 male pools were captured in BG-Sentinel traps.

Weather data is reported in the supplementary online material (S1 Table). Throughout the study period, wind speed ranged from 0.45 m/s to 7.16 m/s with a net wind speed of 1.84 m/s. Wind direction ranged from -23.8 to 325.7 with a net wind direction of 150.1 vector degrees, equating to a south by southeast wind direction. Cumulative rainfall from June to August 2013 is 71.9 mm.

### *Cx*. *quinquefasciatus* dispersal

**Fig 9:**
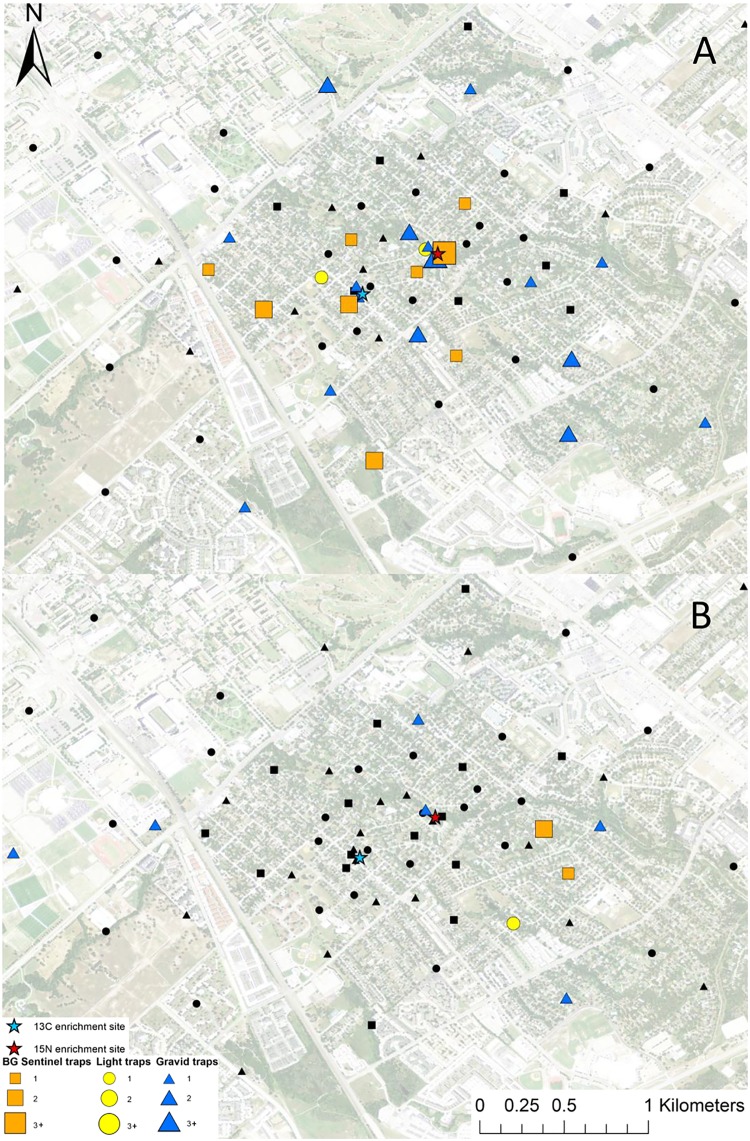
Map of mark-capture study region in College Station, Texas demonstrating the ^13^C and ^15^N-enrichment larval habitat and the locations of captured of marked *Cx*. *quinquefasciatus* females (A), and *Cx*. *quinquefasciatus* males (B). Trap symbols that are black captured zero marked pools and numbers next to symbols in legend represents the number of marked pools captured. The map image was published by the USDA Aerial Photography Field Office as part of the National Agriculture Imagery Program (NAIP), and downloaded as a GIS file. Figure was produced using ArcGIS 10.2 (Esri, Redlands, CA).

A total of 2,066 female *Cx*. *quinquefasciatus* pools (8,002 individuals) were analyzed for presence of stable isotope enrichment. Of those tested, 12 were enriched with ^15^N with a mean δ^15^N of 1,273.6±530.0 ‰ (Figs 1 and 9A). The mean δ^15^N of unenriched female *Cx*. *quinquefasciatus* mosquito pools was 9.4±0.1 ‰. We estimate a re-capture rate of 2.9% for *Cx*. *quinquefasciatus* that emerged from ^15^N. The mean distance traveled (MDT) for female *Cx*. *quinquefasciatus* marked with ^15^N was 0.4 km. The closest trap with a captured marked mosquito was 26.6 m and the furthest was 596.7 m from the ^15^N enrichment site (Fig 1). Of the 2,066 female *Cx*. *quinquefasciatus* pools analyzed, 28 were enriched with ^13^C with a mean δ^13^C of 23.1±5.4 ‰ (Figs 2 and 9A). Mean δ^13^C of unenriched female *Cx*. *quinquefasciatus* pools was -22.1±0.1 ‰. We estimated a re-capture rate of 10.0% for *Cx*. *quinquefasciatus* that emerged from ^13^C. The MDT for female *Cx*. *quinquefasciatus* that emerged from ^13^C was 1.0 km. The nearest trap with a marked mosquito was 27.7 m and the furthest was 1.9 km from the ^13^C enrichment site (Fig 2).

A total of 161 male *Cx*. *quinquefasciatus* pools (632 individuals) were analyzed for stable isotope enrichment. Of these, one was enriched with ^15^N with a mean δ^15^N of 685.8 ‰ (Figs 3 and 9B). The mean δ^15^N of unenriched male *Cx*. *quinquefasciatus* mosquito pools was 8.1±0.2 ‰. The estimated re-capture rate was 0.2% for male *Cx*. *quinquefasciatus* that emerged from the ^15^N enrichment site. The MDT for male *Cx*. *quinquefasciatus* that emerged from ^15^N was 0.3 km. The only trap with a captured marked mosquito was 64.1 m from the ^15^N enrichment site (Fig 3). Of the 161 male *Cx*. *quinquefasciatus* pools tested, nine were enriched with ^13^C with a mean δ^13^C of 58.2±10.9 ‰ (Figs 4 and 9B). The mean δ^13^C of unenriched male *Cx*. *quinquefasciatus* mosquito pools was -22.3±0.2 ‰. The estimated re-capture rate was 3.2% for male *Cx*. *quinquefasciatus* that emerged from the ^13^C enrichment site. The MDT for male *Cx*. *quinquefasciatus* that emerged from ^13^C enrichment site was 1.2 km. The nearest trap with a captured marked mosquito was 0.84 km and the furthest was 1.7 km from the ^13^C enrichment site (Fig 4). Mean distance traveled and sample sizes for total and marked pools for both sexes of *Cx*. *quinquefasciatus* are summarized in Table 1.

**Table 1:** Mean distance traveled (MDT) in kilometers +/- standard error for *Cx*. *quinquefasciatus* and *Ae*. *albopictus* based on sex and stable isotope enrichment type.

Sex	Isotope	Species	MDT (km)	N _pools_	N _marked pools_
Female	^13^C	*Cx*. *quinquefasciatus*	1.0 ± 0.4	2,066	28
		*Ae*. *albopictus*	0.4 ± 0.0	692	13
	^15^N	*Cx*. *quinquefasciatus*	0.4 ± 0.0	2,066	12
		*Ae*. *albopictus*	0.3 ± 0.0	692	16
Male	^13^C	*Cx*. *quinquefasciatus*	1.2 ± 0.2	161	9
		*Ae*. *albopictus*	1.1 ± 0.1	170	12
	^15^N	*Cx*. *quinquefasciatus*	0.3 ± 0.0	161	1
		*Ae*. *albopictus*	0.3 ± 0.0	170	2

The best-fit model predicting the probability of a marked pool of *Cx*. *quinquefasciatus* female mosquitoes incorporated an interaction between distance from the enrichment site and trap type (weight = 0.56). The best-fit model was 2.8-times more likely than a nested model that excluded the interaction term (ΔAICc = 2.1, weight = 0.20), 5.1-times more likely than a model that only included distance from source (ΔAICc = 3.3, weight = 0.14), and 7.0-times more likely than the full model that also included direction of dispersal as a fixed effect (ΔAICc = 3.9, weight = 0.08; Table 2). In general, models with a fixed effect of direction and the intercept only model (ΔAICc = 10.7, weight ≈ 0) fit the data poorly (Table 2). The interaction in the best-fit model suggested that the probability of capture declined with distance, but the decline was steeper for pools of individuals from host seeking relative to oviposition traps. *Cx*. *quinquefasciatus* males dispersed slightly further than females, however the interaction effect between sex and distance was not significant (p = 0.18; parametric bootstrap of the log-likelihood ratio).

**Table 2:** Summary of nested models and corresponding Akaike information criteria.

Models	AICc	ΔAICc	DF	Weight
a) *Culex quinquefasciatus*				
dist, trap type, dist*trap type	442.4	0	7	0.56
dist, trap type	444.5	2.1	6	0.20
dist	445.6	3.3	5	0.11
dist, trap type, direction, dist*trap type	446.3	3.9	10	0.08
dist, trap type, direction	448.3	5.9	9	0.03
dist, direction	448.9	6.5	8	0.02
intercept-only	453.0	10.7	4	0.00
trap type	453.7	11.4	5	0.00
direction	455.2	12.8	7	0.00
b) *Aedes albopictus*				
dist	202.4	0	5	0.89
dist,direction	206.6	4.2	8	0.11
direction	239.1	36.7	7	0.00
intercept-only	251.6	49.2	4	0.00

*dist = distance from larval habitat, trap type has 2 levels (host-seeking or oviposition), direction has four levels (northwest, northeast, southwest, southeast), “*” denotes an interaction

**Fig 10:**
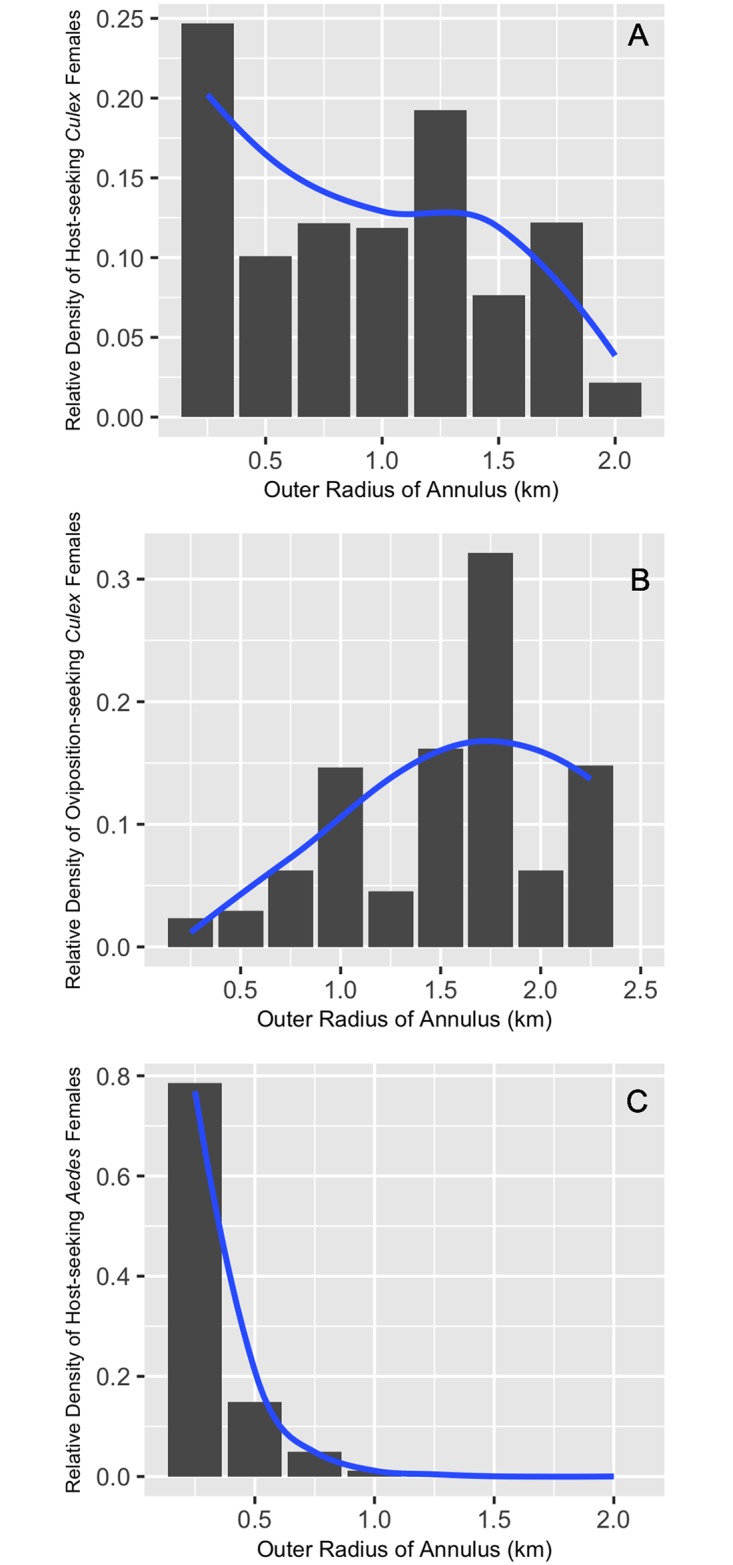
Estimates of the relative density of marked female mosquitoes corrected for trap effort and annulus area for each 250 meter annulus in the analysis. Blue lines represent approximate average curves for the various estimates of relative density across the annuli. The curve was estimated with the smoother function in ggplot2 (geom_smooth). Subpanels A, B, and C correspond to estimated densities of host-seeking *Cx*. *quinquefasciatus* females, oviposition-seeking *Cx*. *quinquefasciatus* females, and host-seeking *Ae*. *albopictus* females, respectively.

The analysis of relative density corrected for area and trap effort estimated that 41% of *Cx*. *quinquefasciatus* females that were host-seeking occurred between 1–2 km from their respective natal site (Fig 10A), while 59% remained within a kilometer of their natal larval habitat. In contrast, 59% of *Cx*. *quinquefasciatus* females that were seeking oviposition sites occurred between 1–2 km away from their natal larval habitat, while 15% occurred greater than 2 km away from their natal site (Fig 10B). Only 26% remained within 1 km of their natal site (Fig 10B).

### *Ae*. *albopictus* dispersal

**Fig 11:**
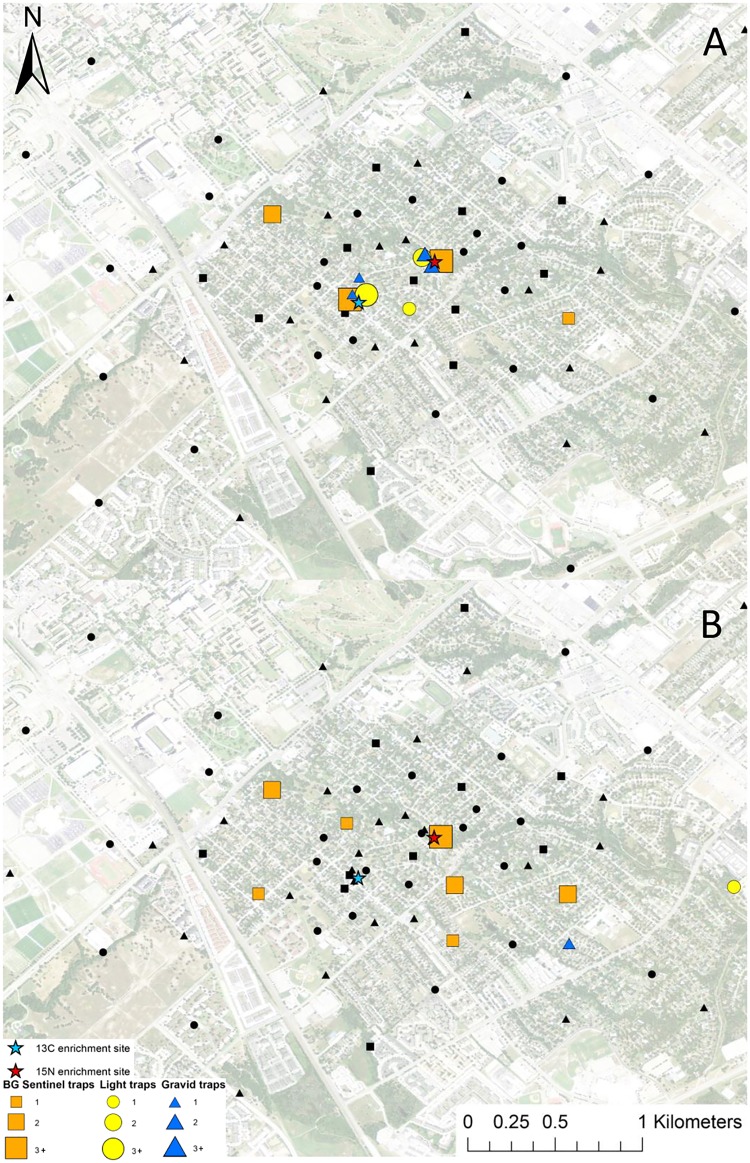
Map of mark-capture study region in College Station, Texas demonstrating the ^13^C and ^15^N-enrichment larval habitat and the locations of captured of marked *Ae*. *albopictus* females (A), and *Ae*. *albopictus* males (B). Trap symbols that are black captured zero marked pools and numbers next to symbols in legend represents the number of marked pools captured. The map image was published by the USDA Aerial Photography Field Office as part of the National Agriculture Imagery Program (NAIP), and downloaded as a GIS file. Figure was produced using ArcGIS 10.2 (Esri, Redlands, CA).

A total of 692 female *Ae*. *albopictus* pools (2,535 individuals) were tested for the presence of ^15^N and ^13^C. Of those tested, 16 were enriched with ^15^N with a mean δ^15^N of 1,388.3±278.0 ‰ (Figs 5 and 11A). The mean δ^15^N of unenriched female *Ae*. *albopictus* mosquito pools was 10.5±0.1 ‰. The estimated recapture rate was 18.0%, for *Ae*. *albopictus* females that emerged from ^15^N enriched sites. The MDT for ^15^N marked female *Ae*. *albopictus* was 0.3 km (Table 1). The closest trap with a captured marked mosquito was 26.6 m and the furthest was 737.5 m from the ^15^N enrichment site (Fig 5). Of the 692 female *Ae*. *albopictus* pools analyzed for stable isotopes, 13 were enriched with ^13^C with a mean δ^13^C of 72.5±29.0 ‰ (Figs 6 and 11A). The mean δ^13^C of unenriched female *Ae*. *albopictus* mosquito pools was -23.0±0.1 ‰. The estimated re-capture rate of ^13^C marked females was 3.8%. The MDT of female *Ae*. *albopictus* that emerged from ^13^C enrichment site was 0.4 km (Table 1). The nearest trap with a captured marked mosquito was 45.3 m and the furthest was 656.2 m from the ^13^C enrichment site (Fig 6).

A total of 170 male *Ae*. *albopictus* pools (671 individuals) were analyzed for stable isotope enrichment. Of these, two were enriched with ^15^N with a mean δ^15^N of 818.8±245.9 ‰ (Figs 7 and 11B). The mean δ^15^N of unenriched *Ae*. *albopictus* mosquito pools was 9.5±0.2 ‰. The estimated re-capture rate was 2.3%. The MDT for male *Ae*. *albopictus* that emerged from ^15^N was 0.3km (Table 1). The only trap with captured marked mosquitoes was 33.5 m from the ^15^N enrichment site (Fig 7). Of the 170 male *Ae*. *albopictus* pools tested for stable isotopes, 12 were enriched with ^13^C and a mean δ^13^C of 89.5±8.0 ‰ (Figs 8 and 11B). The mean δ^13^C of unenriched male *Ae*. *albopictus* pools was -23.5±0.1 ‰. The estimated re-capture rate was 3.5%. The MDT for male *Ae*. *albopictus* that emerged from the ^13^C enrichment site was 1.1 km (Table 2). The nearest trap location with a captured marked individual was 314.1 m and the furthest was 1.9 km from the ^13^C enrichment site (Fig 8). Mean distance traveled and sample sizes for total and marked pools for both sexes of *Ae*. *albopictus* are summarized in Table 1.

**Fig 12:**
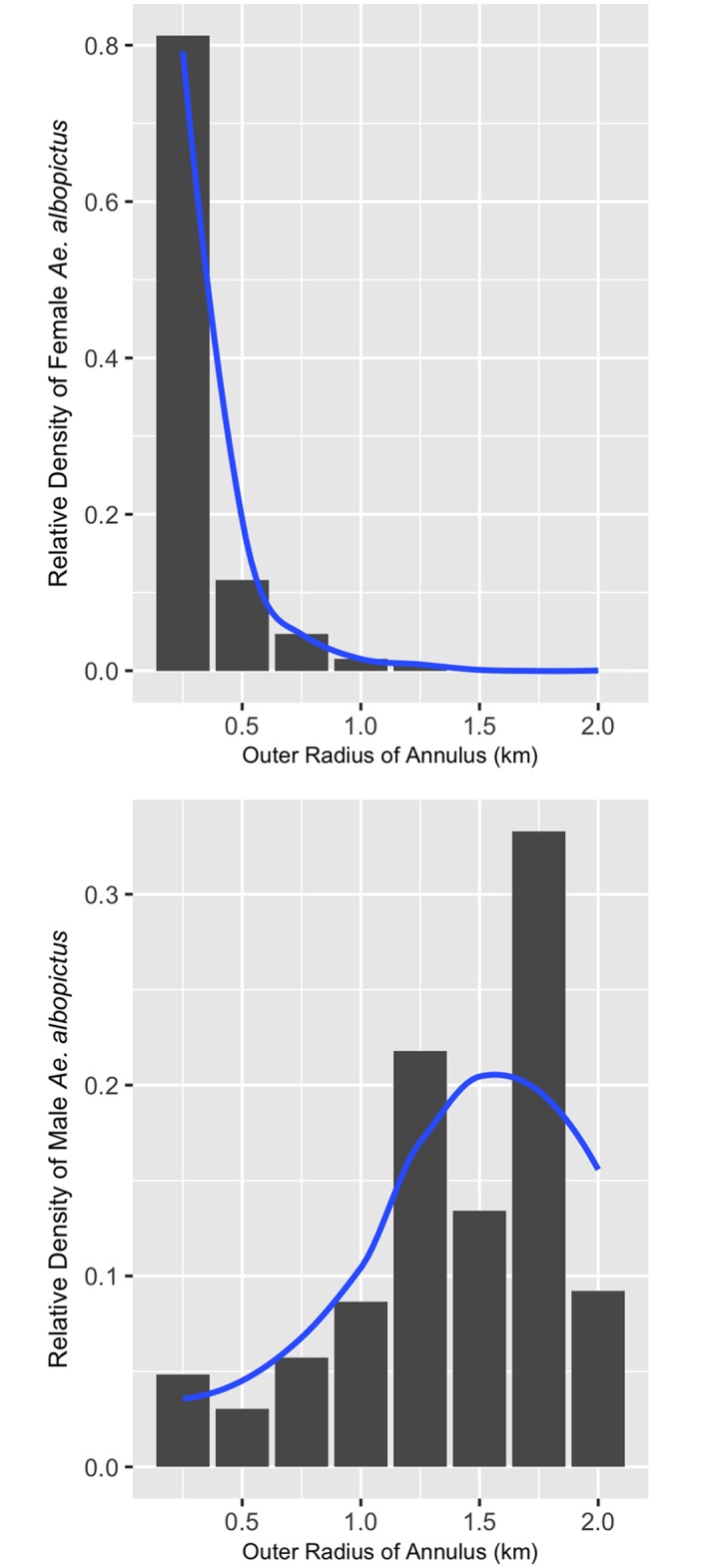
Estimates of the relative density of marked female and male *Ae*. *albopictus* corrected for trap effort and annulus area for each 250-meter annulus in the analysis from a model that explored an interaction between sex and distance on the probability of capture (see methods). Blue lines represent approximate average curves for the various estimates of relative density across the annuli. The curve was estimated with a smoother function in ggplot2 (geom_smooth).

The model predicting the probability of enrichment in female *Ae*. *albopictus* pools as a function of distance from the source (weight = 0.89) better fit the data than models that included direction of dispersal (Table 2) and the intercept-only model (ΔAICc = 49.2, weight ≈ 0; Table 2). The sex of individual *Aedes albopictus* interacted with distance from the source to affect the probability of an enriched pool (p = 0.001; parametric bootstrap of the log-likelihood ratio), with model parameters suggesting that that males disperse farther than females (Fig 12).

The analysis of relative density corrected for area and trap effort estimated that 100% of *Ae*. *albopictus* females that were host-seeking occurred within 1 km from their respective natal site (Fig 10C), with 79% occurring within 250m.

## Discussion

Our study quantified the dispersal patterns of two medically important mosquito vectors, *Cx*. *quinquefasciatus* and *Ae*. *albopictus*, for both sexes at the same location, simultaneously. Thus our protocol, rare in the mosquito dispersal literature [8], is particularly powerful for the direct comparison of dispersal patterns between mosquito species and sexes. Such comparisons are integral to the development of effective vector control strategies by guiding the optimization of spatial scale for intervention campaigns that may target different vector-pathogen systems. Here, we discuss these results and focus on how they may influence the development of general vector control strategies to control emerging mosquito-borne pathogens.

Our data reveal that the dispersal patterns of *Cx*. *quinquefasciatus* and *Ae*. *albopictus*, two mosquitoes whose global ranges broadly overlap, are fundamentally different. Similar to other studies, we show that many *Cx*. *quinquefasciatus* females disperse 1–2 kilometers, while most *Ae*. *albopictus* females remain within 300m of the larval habitat from which they emerged. Long-distance dispersal decreases the efficacy of strategies to contain outbreaks and undermines intervention strategies [3]. In addition, long-distance dispersal increases the spatial scope of vector control efforts, and increases the odds that successfully treated areas may be reinfested from non-treated areas. Our data suggest that outbreaks of mosquito-borne disease will be inherently more difficult to control if vectored by *Cx*. *quinquefasciatus* relative to *Ae*. *albopictus* and other short distance dispersers like *Ae*. *aegypti*. This highlights the potential for *Cx*. *quinquefasciatus* and other closely related *Culex* mosquitoes to drive the emergence of vector-borne infectious disease for pathogens when their vectorial capacity is high.

We find evidence that male *Ae*. *albopictus* dispersed farther than females. Previous studies have found no difference between the dispersal distances amongst the sexes in *Ae*. *aegypti*, or that females tended to disperse farther than males. Inbreeding avoidance and the reduction in competition among kin for local resources may drive the evolution of sex-biased dispersal [49, 50], as dispersing individuals are less likely to mate with a relative or to compete for mates and other resources with brothers or sisters. It may be more advantageous for males to disperse from natal sites when females are philopatric. This may explain why we observed a strong bias toward long-distance male dispersal in *Ae*. *albopictus* compared to *Cx*. *quinquefasciatus*. Future studies should continue to address sex-biased dispersal in mosquitoes. The stable isotope protocol employed here may be particularly useful as it allows males and females to disperse naturally from their natal site. This facilitates comparison between sexes among individuals that are emerging from the same place at approximately the same time, reducing the potential for landscape features or weather patterns to influence mosquito dispersal and produce artifacts. With the advent of genetically-modified sterile or autocidal male mosquitoes as next-generation vector control strategies, understanding male dispersal patterns among target mosquito species in different environmental and ecological contexts will be crucial to the field application and ultimate success of these techniques [51].

While our data has broad applicability to vector behavior and control, it is particularly relevant to Texas, a region with a relative high burden of mosquito-borne viruses and a relatively small mosquito control community [29] arboviorus transmission that is woefully understudied. Texas is endemic with WNV since 2002 and large epidemics of WNV neuroinvasive disease occurred in Dallas, TX in 2012 [52] and Houston, TX in 2014 [53]. Previously, *Ae*. *albopictus* in the United States was primarily regarded as a nuisance mosquito. However due to epidemic dengue transmission in parts of the Texas, Florida, and Hawaii [36, 54, 55] and the introduction of CHIKV to the United States in 2014 [55], public health officials are being encouraged to develop effective control strategies for *Ae*. *albopictus* and *Ae*. *aegypti*. Indeed, the first case of autochthonous CHIKV in Texas occurred in 2015 in Cameron County (Texas Department of State Health Services). In addition, the ZIKV epidemic in Latin America and its probable future emergence in Texas have only increased the urgency to devise integrated vector control strategies toward these mosquito vectors. Such efforts will mitigate the potential for these diseases to establish in Texas, and as a result reduce the potential for unimpeded human movement to spread mosquito-borne disease within the United States.

Isotopic enrichment of larvae is simple, effective, relatively inexpensive, and can be achieved in the field. Based on the limited controlled studies, stable isotope marking of the insect does not inhibit the growth or normal biology, and offers life-long retention. However, the method has some limitations. The stable isotope analysis is expensive and can range from $5 to much higher per sample depending on the isotope, facility, and the turn-around time. In the current study we analyzed a total of 3,089 at $6 per sample for a total cost of $18,534. In addition, we analyzed 20 immature mosquitoes for ^15^N and another 20 for ^13^C which was $1,200 ($30 per sample for rush turn-around time at Isotech Laboratories Inc.). Additionally, this technique requires a long delay between mosquito collection in the field and eventual stable isotope analysis. From the time of sample receipt, stable isotope labs vary in their turn-around time of results from about 1 week to 20 weeks. Extreme attention to detail is necessary for sample and database management to ensure the stable isotope results are accurately matched to the field data. Continued use of stable isotope analysis in both enrichment and natural abundance study could eventually reduce the cost of this technique and technological advances in the technique could offer more rapid turnaround. Temporal dispersal measurements are often difficult to obtain using this technique, as mosquitoes are not released at a single time point. As a result of this study design, it is difficult to quantify the effects that abiotic factors such as temperature, precipitation, wind, and humidity have on dispersal when using stable isotopes as a marker. This is due to the challenge of determining the exact age of the marked mosquito or the date of the dispersal event. One other challenge with most studies of mosquito dispersal is that the trap design can influence the results of the study; Guerra et al. [8] found a positive correlation between the radius of the experimental area with traps and the mean distance traveled. In the present study, we deployed traps out to about 2 km but the density in each annuli was not constant and the trap types were not balanced among annuli, which could have influenced our results. Stable isotope marking has recently been demonstrated for mosquito vectors of malaria in Tanzania [56] and studies are underway to use this technique not to study dispersal per se, but to evaluate the success of a novel intervention strategy. This study in Tanzania and the current study in Texas reveal the diverse ways in which stable isotopes can be used as useful tools to study of mosquito biology in diverse settings.

## Supporting information

S1 Table Monthly mean maximum and minimum temperature, relative humidity, rainfall, wind speed and wind direction for June, July and August 2013 in College Station, TX. (DOCX)
