# Supplementary material for: Dispersal of male and female Culex quinquefasciatus and Aedes albopictus mosquitoes using stable isotope enrichment
Source: PLoS Negl Trop Dis. 2017 Jan 30;11(1):e0005347. doi: 10.1371/journal.pntd.0005347 (PMC5300284; doi:10.1371/journal.pntd.0005347)
Supplement: S1 Table — (DOCX) [file pntd.0005347.s001.docx]

Supplemental Table 1. Monthly mean maximum and minimum temperature, relative humidity, rainfall, wind speed and wind direction for June, July and August 2013 in College Station, TX.

|  | Mean | Mean | Mean Relative | Mean Rainfall | Net Wind | Net Wind Direction |
| --- | --- | --- | --- | --- | --- | --- |
| Month | MaxTemp (°C) | MinTemp(°C) | Humidity (%) | (mm.) | Speed (m/s) | (degrees) |
| June | 33.9 ± 0.4 | 22.9 ± 0.4 | 36.6 ± 1.3 | 1.3 ± 1.1 | 2.0 | 138.49 |
| July | 33.8 ± 0.5 | 23.4 ± 0.3 | 35.1 ± 2.0 | 0.9 ± 0.5 | 1.83 | 153.04 |
| August | 35.6 ± 0.4 | 24.0 ± 0.2 | 31.1 ± 1.3 | 0.6 ± 0.4 | 1.78 | 159.56 |
